# A Moderated Mediation Model of AI-Driven Identity Threats and Employee Cyberloafing: The Role of AI-Inclusive Identity

**DOI:** 10.3390/ejihpe16040052

**Published:** 2026-04-01

**Authors:** Alqa Ashraf, Qingfei Min, Aleena Ashraf

**Affiliations:** School of Economics and Management, Dalian University of Technology, Dalian 116024, China; minqf@dlut.edu.cn (Q.M.); aleena@mail.dlut.edu.cn (A.A.)

**Keywords:** professional identity threat, cyberloafing, loss of autonomy, loss of skill, human–AI collaboration

## Abstract

This study intended to examine how human–AI collaboration-based identity threat appraisals in the form of the loss of autonomy and loss of skill trigger a professional identity that fosters cyberloafing. Based on social identity theory, this study applied a three-wave survey design with 507 employees. The proposed research model was tested using partial least squares structural equation modeling (PLS-SEM) with SmartPLS 4, which enabled the assessment of both measurement and structural models. The perceived loss of skill and loss of autonomy are positively associated with professional identity threat, which mediates their relationships with cyberloafing. AI-inclusive identity weakens these associations for the loss of autonomy, suggesting that employees with strong AI-inclusive identity exhibit weaker professional identity threat. When integrating AI, organizations should mitigate appraisals of the loss of autonomy and loss of skill through participatory design, role redesign, and communication that emphasizes unique human contributions. Supporting healthy AI–human identity integration may reduce counterproductive behaviors such as cyberloafing. By positioning identity threat appraisals as human–AI collaboration-driven antecedents of professional identity threat and cyberloafing, this study extends social identity theory to human–AI contexts. It further demonstrates that over-identification with AI may heighten professional identity threats by diminishing the value of uniquely human contributions.

## 1. Introduction

Human–AI collaboration describes the contact and cooperation between employees and AI systems for completing job tasks more effectively (Kong et al., 2023). This interaction has been found to shape employees’ behaviors and performance in notable ways (Ma et al., 2024; Budhwar et al., 2022). On the positive side, AI adoption encourages innovative behavior and improves service quality (Yan & Teng, 2025; Duan et al., 2024). Yet it can also lead to negative outcomes such as disengagement from work and counterproductive work behaviors (Meng et al., 2025; Bai & Zhang, 2025). Some scholars caution that employees may exploit the ease of AI to complete tasks superficially, thereby decreasing their effort and increasing laziness during working hours (Saluja et al., 2024). One demonstration of this is cyberloafing, a form of non-work-related online behavior that employees engage in during office hours (Gupta et al., 2025). Cyberloafing includes activities such as browsing e-commerce platforms or sending personal messages during work time (Hessari et al., 2025; Askew et al., 2014). Past research has shown that cyberloafing weakens employee engagement and diminishes productivity (Tsai, 2023; Hessari et al., 2025).

It is vital to recognize that collaboration between human and AI highlights the corresponding strengths of both human and AI systems (Jia et al., 2024; Kong et al., 2023). Unlike traditional workers, employees collaborating with AI can achieve higher-quality outcomes and greater efficiency, resulting in performance that surpasses that of conventional work arrangements (Seeber et al., 2020). However, despite these performance gains, collaboration with AI can also undermine employees’ sense of professional identity by challenging their autonomy and perceived competence, thereby weakening their identification with their occupational role. An individual’s need for autonomy and competence is essential for psychological well-being and motivation; when AI assumes decision-making authority or handles skill-intensive tasks, these core needs may be undermined (Deci & Ryan, 2000; Alfrey et al., 2023). These experiences can be framed as identity threat appraisals, where employees perceive AI collaboration not only as a productivity-enhancing tool but also as a team member with potential to threaten their professional identity. It is this sustained state of professional identity threat that often encourages employees to engage in counterproductive surviving mechanisms to preserve self-esteem, such as cyberloafing (Askew et al., 2014). 

Despite growing interest in human–AI collaboration, existing research has largely examined its consequences through lenses such as organizational performance, productivity, and technostress, offering limited insight into the identity-based processes through which AI-driven work transformations shape employee behavior (Budhwar et al., 2022; Derks et al., 2015). In particular, little is known about how employees cognitively appraise AI-induced changes in autonomy and skills as threats to their professional identity or how such appraisals translate into subtle forms of disengagement such as cyberloafing. Moreover, prior studies tend to treat employee reactions to AI as relatively uniform, overlooking individual differences that may condition identity-based responses to AI-enabled work arrangements (Raisch & Krakowski, 2021). Addressing these gaps is critical for developing a more nuanced understanding of why human–AI collaboration produces divergent behavioral outcomes and for identifying when human–AI collaboration undermines, rather than supports, sustainable employee engagement. So, this study aims to answer the following question:

RQ: How do human–AI collaboration-based identity threat appraisals, in the form of the perceived loss of skill and autonomy, contribute to professional identity threats and subsequently influence employees’ cyberloafing behaviors?

Grounded in social identity theory, this study examines how the AI-driven loss of autonomy and loss of skill influence cyberloafing through professional identity threat. Using a three-wave survey (*N* = 507), the results show that both the loss of autonomy and loss of skill increase cyberloafing by heightening professional identity threat. Moreover, AI-inclusive identity moderates this process by weakening the identity-based pathway, thereby weakening the indirect effects at higher levels of AI-inclusive identity. Theoretically, this study extends social identity theory to human–AI collaboration, establishes professional identity threat as a key mechanism underlying counterproductive behavior, and clarifies the boundary role of AI-inclusive identity. Practically, the findings suggest that organizations can reduce cyberloafing by safeguarding employee skills and autonomy and by fostering constructive identity integration with AI.

## 2. Theorical Foundation and Hypotheses

### 2.1. Social Identity Theory in the Era of AI

Unlike earlier generations of information technologies that primarily augmented task execution, AI systems increasingly replicate or substitute core professional functions such as judgment, problem-solving, and decision-making. In healthcare, for example, AI systems like IBM Watson redefine the physician’s diagnostic role by providing rapid data analysis, which expands autonomy by freeing time for complex case reviews but can also constrain it by creating a new imperative to justify decisions that deviate from AI recommendations (Jiang et al., 2017). In finance, JPMorgan Chase’s COiN platform transforms legal analysts into validators and negotiators while simultaneously constraining their autonomy by embedding the AI platform’s output as a procedural standard that must be actively accepted (Davenport et al., 2018). This dynamic demonstrates a broader trend: AI fundamentally alters job architecture. In creative industries, tools like Adobe Firefly redefine artists as art directors and curators, expanding creative possibilities while introducing new constraints as they must work within AI’s technical and stylistic parameters (Brynjolfsson & McAfee, 2014). In customer service, chatbots from companies like Zendesk shift human agents into roles of emotional and critical problem-solvers, granting them more complex interactions but constraining their control over the standardized initial customer triage process (Echegu, 2024). Even in manufacturing, collaborative robots like Tesla’s Optimus redefine the role of assembly line workers as encompassing system oversight only while tightly coupling their workflow to the robot’s pace and capabilities. These examples underscore a fundamental shift: AI is no longer just a tool but a team member which can significantly influence an employee’s professional identity. While previous technological revolutions altered job structures and work processes, they rarely encroached upon employees’ core professional identities to the same extent as AI, which increasingly performs evaluative and cognitive functions traditionally reserved for human expertise. This occurs as humans engage in social identification processes with their hybrid teams, where the capabilities and role of AI can reshape the perceptions of one’s own tasks, skills, and value (Ashforth & Mael, 1989). Employees appraise these changes as threats to who they are as professionals, rather than simply to how tasks are performed (Petriglieri, 2011). 

These threat appraisals give rise to professional identity threat (Mirbabaie et al., 2022), stress, anxiety, and emotional exhaustion (Ellemers et al., 2002; Liu & Geertshuis, 2019). As an outcome, employees may have one of the following three responses: the first consists of defensive behaviors such as withdrawal, silence, and the avoidance of identity-relevant tasks to protect the self from further harm (Ashforth & Mael, 1989; Ellemers et al., 2002; von Hippel et al., 2024); the second is represented by proactive behaviors, such as job crafting, upskilling, and identity reconstruction (Ibarra, 1999; Petriglieri, 2011); and the third is coping behavior such as identity distancing and compensatory activities (Breakwell, 1986). Compensatory activities may include cyberloafing, rule-bending, or other counterproductive actions, as emotional exhaustion in the form of threatened identity weakens self-regulatory capacity (Liu & Geertshuis, 2019). 

Because AI systems are often perceived as non-negotiable and embedded in organizational infrastructures, employees experiencing identity threat may resort to covert withdrawal behaviors such as cyberloafing rather than overt resistance. These behaviors function less as intentional misconduct and more as compensatory mechanisms through which employees attempt to restore autonomy, control, or emotional balance under identity strain. Prior cyberloafing research (presented in Table 1) has been dominated by resource/strain explanations and has treated cyberloafing primarily as a deviance/withdrawal response. For example, COR-based models show that role stress predicts cyberloafing through emotional exhaustion and resource loss processes (Lu et al., 2024). They conceptualize cyberloafing as an avoidant coping strategy that provides short-term psychological distance from demanding work (Varghese & Barber, 2017). These explanations are powerful, but they typically remain resource-centric: they explain, “I’m depleted, so I disengage,” without specifying why a digital/AI change is experienced as existentially threatening for professionals.

Moreover, much of the literature frames autonomy loss through control/compliance or fairness lenses, rather than as a threat to “who I am as a professional” (Schlund & Zitek, 2024). This study connects these two streams via an identity mechanism: digital/AI-driven skill obsolescence and autonomy loss are not just “stressors” but identity-relevant appraisals that undermine competence- and agency-based professional self-definitions. Identity threat work argues that identity threats are appraisals that a situation could devalue an identity, alter its meaning, or constrain its enactment, hence prompting coping and defensive responses (Freeburg & Klein, 2025; Petriglieri, 2011). By modeling professional identity threat as the central mediator, this study answers why technologically induced skill and autonomy loss generate not only strain but also self-protective coping behaviors such as cyberloafing. 

### 2.2. Development of Hypotheses

#### 2.2.1. AI-Driven Identity Threat Appraisals and Cyberloafing

Professional identity is formed through individuals’ self-definition in terms of the meanings, values, and norms associated with their professional role (Ashforth & Mael, 1989; Ibarra, 1999). Employees working with artificial intelligence may perceive that the values and meanings underlying their professional roles are under threat, as AI systems encroach upon tasks traditionally defining human expertise while simultaneously altering the level of control they exercise over their work (Petriglieri, 2011). These perceptions are referred to as identity threat appraisals, which are activated when individuals perceive that the continuity, distinctiveness, or value of their role-based identity is being undermined (Lazarus & Folkman, 1984; Swann et al., 2010). 

In the context of human–AI collaborative work settings, employees may perceive that their skills are no longer needed because AI systems increasingly outperform in tasks that previously signaled human competence, expertise, and professional value (Raisch & Krakowski, 2021). Perceptions of deskilling and reduced human contribution due to AI can undermine employees’ sense of usefulness, prompting disengagement and withdrawal-oriented coping behaviors (Tarafdar et al., 2019; Ragu-Nathan et al., 2008). Cyberloafing, in particular, has been widely conceptualized as a discretionary, low-risk withdrawal behavior through which employees cope with perceived threats or frustrations arising from work technologies (Lim & Teo, 2005; Lim & Chen, 2012). Recent studies further indicate that when artificial intelligence is appraised as threatening employees’ skills or value, individuals are more likely to disengage from work tasks and redirect attention toward non-work online activities (Derks et al., 2015; Q. Zhang et al., 2025). So, this study proposed the following hypothesis:

**H1a.**  *The AI-driven loss of skill is positively associated with cyberloafing.*

Similarly, when employees work alongside artificial intelligence, they may feel a loss of autonomy as AI systems increasingly structure, guide, and constrain how tasks are performed, thereby reducing employees’ discretion and control over work processes (Kellogg et al., 2020; Raisch & Krakowski, 2021). When employees experience diminished control, they may seek alternative means to reassert personal agency and restore a sense of power in their work environment (Deci & Ryan, 2000; Parker & Grote, 2022). Cyberloafing can be understood as one such power-restorative behavior, through which employees reclaim control by engaging in self-directed, non-work online activities that lie outside task prescriptions (Lim, 2002; Pindek et al., 2018). Autonomy-reducing algorithmic systems may trigger subtle resistance behaviors such as cyberloafing, as employees attempt to reclaim a sense of control and agency within tightly monitored digital environments (Carter & Grover, 2015; Kellogg et al., 2020). Taken together, this literature suggests that the AI-driven loss of autonomy is likely to increase cyberloafing as a means of regaining personal power and control at work. So, the following hypothesis is proposed.

**H1b.**  *The AI-driven loss of autonomy is positively associated with cyberloafing.*

#### 2.2.2. Mediating Role of Professional Identity Threat

Specialized skills and autonomy over work decisions are core components of one’s professional identity, which signal professional competence, jurisdiction, and control over one’s role (Freidson, 2001; Pratt et al., 2006). Professionals derive their sense of worth and role legitimacy from the recognition of their expertise and their discretion in applying it, such that threats to skills or autonomy are appraised as direct challenges to professional identity rather than as mere task-level disruptions (Petriglieri, 2011; Barley et al., 2017). 

Employees collaborating with AI systems that augment or outperform human expertise perceive a loss of skill insofar as tasks that previously signaled professional competence and distinctiveness become less central or less valued (Raisch & Krakowski, 2021). Professional identity is closely tied to the possession and recognition of specialized knowledge and skills, such that threats to perceived expertise undermine individuals’ sense of professional worth and legitimacy (Pratt et al., 2006; Petriglieri, 2011). Perceptions of deskilling or the devaluation of human expertise are associated with heightened concerns about professional relevance and identity erosion (Barley et al., 2017; Derks et al., 2015). Accordingly, when AI-driven work arrangements are appraised as eroding employees’ skills, such appraisals are likely to translate into heightened professional identity threat. So, the following hypothesis is proposed:

**H2.**  *The loss of skill is positively associated with professional identity threat.*

In addition to skills, professional identity is deeply rooted in autonomy, discretion, and control over work decisions, which signal professional status and role ownership (Freidson, 2001; Parker & Grote, 2022). AI systems that algorithmically guide, evaluate, or constrain work processes may reduce employees’ decision freedom, thereby altering their perceived role authority and professional standing (Kellogg et al., 2020). Such reductions in autonomy are often interpreted not merely as efficiency-enhancing controls but as challenges to employees’ professional judgment and legitimacy (Meijerink & Bondarouk, 2023; Möhlmann et al., 2021). Because autonomy constitutes a core symbolic marker of professional identity, its erosion is likely to provoke concerns about diminished role ownership and professional self-definition. Thus, AI-driven autonomy loss is expected to heighten professional identity threat. So, we proposed the following hypothesis:

**H3.**  *The loss of autonomy is positively associated with professional identity threat.*

Employees experiencing professional identity threat disengage psychologically from work contexts that no longer affirm their professional identity (Ashforth & Schinoff, 2016). Identity-threatening work environments are particularly conducive to covert disengagement behaviors, as employees seek to preserve self-integrity while avoiding direct resistance (Lim & Chen, 2012). Integrating this argument into this study, professional identity threat represents a central psychological mechanism through which AI-driven identity threat appraisals translate into employee behavioral responses. The loss of skill and loss of autonomy do not merely alter task characteristics; rather, they challenge fundamental elements through which employees define their professional selves. Social identity theory suggests that when identity-relevant appraisals threaten the self-concept, individuals are motivated to regulate exposure to the threatening context, often through withdrawal or disengagement behaviors (Ashforth & Mael, 1989; Petriglieri, 2011). In AI-enabled workplaces, cyberloafing offers a practical avenue for such disengagement by allowing employees to reclaim psychological distance from work environments perceived as undermining their professional identity. Thus, professional identity threat is expected to mediate the relationships between AI-driven loss appraisals and cyberloafing. So, the following mediated hypotheses are proposed:

**H4.**  *Professional identity threat mediates the relationship between the loss of skill and cyberloafing.*

**H5.**  *Professional identity threat mediates the relationship between the loss of autonomy and cyberloafing.*

#### 2.2.3. The Moderating Role of AI-Inclusive Identity

AI-driven appraisals of the loss of skill and autonomy are not experienced uniformly across employees. Individuals differ in how they interpret these changes depending on the extent to which the use of AI becomes incorporated into their work-related self-concept. Employees who view AI as a collaborative partner and integrate it into their professional role are more likely to perceive AI as an augmentation of their capabilities rather than a threat (Faraj et al., 2018; Jarrahi, 2018). They incorporate AI collaboration into their professional story, sense of mastery, and vision for future development. Based on identity theory, this study argues that when individuals strongly identify with an entity that is implicated in a change, threats originating from that entity are processed as more self-relevant and consequential (Ashforth & Mael, 1989; Petriglieri, 2011). Accordingly, employees with strong AI-inclusive identity may experience a greater salience of autonomy and skill changes, because AI directly occupies a central place in their work-related self-definition. 

Employees with strong AI-inclusive identity view the use of artificial intelligence as an extension of professional capability. This orientation involves developing competencies such as interacting effectively with AI systems, critically evaluating AI-generated outputs, and integrating AI tools into professional workflows (Dell’Acqua et al., 2023). It moves beyond basic digital literacy to encompass a meta-cognitive layer where professionals must learn to collaborate with systems to extend their cognitive and creative reach (Raisch & Krakowski, 2021). Thus, when AI outperforms or replaces tasks central to human expertise, those who identify strongly with AI may be aware of their collaborative (with AI) contribution, thereby weakening its impact on professional identity threat. Similarly, regarding the loss of autonomy, employees with strong AI-inclusive identity may more readily accept algorithmic authority as legitimate and pervasive. This involves internalizing a role (as a supervisor, trainer, and co-creator) with AI systems, actively arranging workflows where judgment and initiative are shared (Raisch & Krakowski, 2021). These professionals exercise autonomy not *from* AI but *through* it (Shneiderman, 2022). This partnership thus redefines autonomy as a collaborative practice, where human agency is expressed in directing, refining, and integrating the capabilities of an AI partner to achieve goals that neither could accomplish as effectively alone (Seeber et al., 2020). Thus, when algorithms undermine autonomy and discretion, they redefine the sense of autonomy of employees with strong AI-inclusive identity, thereby weakening its impact on professional identity threat. Thus, AI-inclusive identity is expected to weaken the positive relationships between AI-driven identity threat appraisals and professional identity threat. So, the following moderating hypotheses are proposed in this study:

**H6.**  *AI-inclusive identity negatively moderates the relationship between the loss of skill and professional identity threat, such that the relationship is weaker when AI-inclusive identity is strong.*

**H7.**  *AI-inclusive identity negatively moderates the relationship between the loss of autonomy and professional identity threat, such that the relationship is weaker when AI-inclusive identity is strong.*

From the prior discussions, it is hypothesized that the interaction between the loss of autonomy and AI-inclusive identity and the interaction between the loss of skill and AI-inclusive identity will affect professional identity threat (Hypotheses 6 and 7). Professional identity threat plays a mediating role between identity threat appraisals and cyberloafing (H2 and H3). Therefore, it is reasonable to predict that the indirect effects of AI-driven identity threat appraisals on cyberloafing will be stronger when employees have a higher level of AI-inclusive identity. So, we hypothesize the following:

**H8.**  *AI-inclusive identity moderates the indirect effects of the loss of autonomy on cyberloafing.*

**H9.**  *AI-inclusive identity moderates the indirect effects of the loss of skill on cyberloafing.*

**Figure 1:**
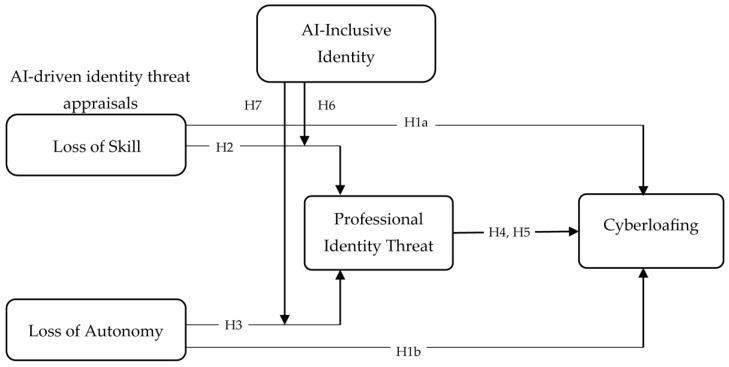
Conceptual model (authors’ own creation).

Figure 1 presents the conceptual model of the study, illustrating the proposed relationships among the key constructs.

## 3. Materials and Methods

### 3.1. Participants and Design

Unlike cross-sectional designs, multi-wave data collection is effective in mitigating common method bias (Gupta et al., 2025). Accordingly, this study employed a three-wave design to enhance the validity of the hypothesized relationships. Respondents were selected through Credamo, an online data collection platform widely used for academic research in organizational and psychological studies (Wu et al., 2024). No restrictions were imposed regarding participants’ geographical location or organizational affiliation. The only eligibility criterion was active engagement in AI collaboration. To ensure the sample accurately reflected this requirement, the survey introduction included the following statement:

“The objective of this research is to understand the cognitive and behavioral responses of employees working in collaboration with AI. Individuals not currently collaborating with AI are kindly requested not to proceed with the questionnaire.”

Participants were also assured of the anonymity of their responses and informed that the data would be used solely for academic research purposes, thereby reducing potential concerns related to privacy or data disclosure.

At Time 1 (6 March 2025), 600 questionnaires were distributed. Respondents provided demographic information and responded to items measuring the AI-driven loss of skill and loss of autonomy. To encourage participation, a small monetary reward (RMB 1) was offered upon completion. After a three-week interval, on 27 March 2025 (Time 2), a follow-up questionnaire was administered to the original participants. This wave focused on assessing participants’ AI- inclusive identity, a multidimensional construct comprising dependence, emotional energy, and relatedness and professional identity threat, another multidimensional construct comprising threat to professional recognition and threat to professional capability. As with the first wave, participants received a small incentive of RMB 1 upon completion. A total of 538 valid responses were collected, resulting in an effective response rate of 89.67%.

Subsequently, on 14 April 2025 (Time 3), the third and final survey was distributed. This questionnaire measured participants’ cyberloafing behaviors following their engagement with AI in the workplace. To acknowledge their continued participation, respondents were compensated with RMB 2.

To ensure data quality, responses were screened for completion time, and cases with implausibly short (<60 s) or excessively long (>300 s) durations were excluded. After this quality check, the final sample comprised 507 valid responses, yielding an overall effective response rate of 84.5%. Descriptive statistics are reported in Table 2.

### 3.2. Measurement Scale

Cyberloafing was assessed using a scale developed by Lim and Teo (2005), which employs a 5-point Likert format ranging from 1 (never) to 5 (very frequently). This scale has consistently demonstrated strong internal reliability across studies (Andel et al., 2019). Participants were asked to indicate the frequency with which they engaged in various non-work-related internet activities during working hours. The following is a sample item: “At work, I send non-work-related e-mail.” In the current study, the scale showed excellent internal consistency, with a Cronbach’s alpha of 0.909.

The loss of skill was assessed using a scale developed by Jussupow et al. (2018), and the usability of this scale was also confirmed by Mirbabaie et al. (2022). Participants were asked to rank their perception of losing expert status and competence at work from 1 = strongly disagree to 5 = strongly agree. The following is a sample item: “due to AI teammate, my specialized work-related skills are not needed anymore.” 

The loss of autonomy measures employees’ sense of having less discretion over how, when, or why they perform their work, and it was assessed using a scale developed by Breaugh (1985). Participants were asked to rank their perception from 1 = strongly disagree to 5 = strongly agree. The following is a sample item: “I cannot choose the way to go about my job, my AI teammate decides that.” 

Threat to professional identity was assessed by adapting a scale by Craig et al. (2019), employing a five-point Likert format from 1 = strongly disagree to 5 = strongly agree. It consists of dimensions comprising threat to professional recognition and capability. The following is a sample item: “Using AI makes me feel discouraged with who I am.”

AI-inclusive identity was measured using an adapted version of the IT identity scale originally developed by Carter and Grover (2015). While IT identity focuses on identification with technology as a work tool, AI-inclusive identity reflects identification with AI as an intelligent, autonomous, and decision-capable collaborator embedded in work processes. This conceptual distinction is supported by Mirbabaie et al. (2022), who validated the relevance of the adapted scale for capturing AI-inclusive identity in workplace contexts. The instrument captures three dimensions—dependence (e.g., “Thinking about myself in relation to the AI, I feel needing it”), emotional energy (e.g., “Thinking about myself in relation to the AI, I feel energized”), and relatedness (e.g., “Thinking about myself in relation to the AI, I feel close to it”). Responses were collected on a 5-point Likert scale ranging from 1 = strongly disagree to 5 = strongly agree. The scale demonstrated strong internal consistency, with a Cronbach’s alpha of 0.918.

To rule out different explanations and account for possible confounding effects, several control variables were included in the analysis. Specifically, age, gender and organizational tenure (in years) were controlled, as these demographic factors have been recognized in prior research as variables that could influence the dependent variables under investigation (Liang et al., 2024).

## 4. Results

This research used SPSS 26.0, AMOS 24.0 and Smart Pls 4 to validate the data. Due to the large number of measurement items associated with the study constructs, multidimensional variables were modeled through internal indicator parceling to enhance indicator quality and improve the overall model fit (Little et al., 2002).

### 4.1. Common Method Bias

Studies involving self-reported data often encounter the critical issue of common method bias (CMB) variance (Schwarz et al., 2017). Podsakoff et al. (2003) describe that this issue arises when data is gathered from a single source, with one respondent providing answers for both dependent and independent variables. To address CMB, this study executed a full collinearity assessment test in Smart PLS, following recommendations from several social science researchers (Mirbabaie et al., 2022). The variance inflation factor (VIF) values in this study were all under the threshold of 3.3, confirming that CMB was not a significant concern (Kock, 2015).

### 4.2. Confirmatory Factor Analysis 

Table 3 shows the model fit indices derived from confirmatory factor analysis using AMOS 24.0. The five-factor model fits the best among all models (RMSEA = 0.078; SRMR = 0.077; CFI = 0.901; TLI = 0.833; IFI = 0.869), which supports discriminant validity among the constructs.

### 4.3. Measurement Model Validation

SmartPLS 3 (Ringle et al., 2020) was used to evaluate the measurement model and test the hypotheses due to the complexity of the research model combined with a small sample size (J. Hair et al., 2016). Reliability was measured using composite reliability (CR) and Cronbach’s alpha (α), with all constructs showing CR and alpha values exceeding the minimum threshold of 0.70 (J. Hair et al., 2016). To determine convergent validity, this study also assessed the average variance extracted (AVE) and factor loadings for all constructs. The AVE results indicated scores above 0.50, meeting the threshold (J. F. Hair et al., 2017), while factor loadings ranged from 0.70 to 0.93, according to standardized values (Cohen, 2013). These findings confirmed strong reliability and convergent validity, as detailed in Table 4. 

### 4.4. Discriminant Validity (DV)

This study employed two primary methods to examine the discriminant validity (DV) of the constructs. First, Fornell and Larcker’s criterion, shown in Table 5, indicated that the square root values of the AVE for each construct were higher than the correlation values in their corresponding rows and columns, confirming DV (Fornell & Larcker, 1981). Second, the heterotrait–monotrait (HTMT) ratio presented in Table 6 showed that all values were below 0.85, indicating no issues (Henseler et al., 2015).

### 4.5. Descriptives and Correlation Coefficients

Table 7 reports the means, standard deviations, and bivariate correlations among the study variables. All constructs exhibit moderate mean values (ranging from 3.31 to 3.39) and adequate variability (SDs between 0.82 and 1.04), suggesting sufficient dispersion for multivariate analysis and no evidence of range restriction. The loss of skill and loss of autonomy are positively correlated with professional identity threat and cyberloafing, AI-inclusive identity is weakly correlated with professional identity threat and cyberloafing, and professional identity threat is negatively correlated with cyberloafing. Overall, the correlation results provide preliminary support for the hypothesized relationships, demonstrate construct distinctiveness, and justify the subsequent PLS-SEM analysis to test mediation and moderation effects.

### 4.6. Testing of Hypotheses

To uncover the complex relationship between variables, main effects, mediating effects and moderating effects are examined. In these analyses, smartPLS 4 estimated the beta values, *t*-statistics and p-values and confidence interval through bootstrap. 

#### 4.6.1. Main Effects

For the main effect analysis, control variables (gender, age and tenure in years), independent variables (loss of skill and loss of autonomy) and dependent variables (cyberloafing) were included in model 1. The results presented in Table 8 show that the loss of skill (β = 0.246, *p* < 0.001) and loss of autonomy (β = 0.340, *p* < 0.001) have a positive and significant influence on cyberloafing. So, hypotheses H1a and H1b are supported, suggesting that both of the identity threat appraisals increase cyberloafing among employees collaborating with AI at their workplace. 

#### 4.6.2. Mediation Effects

For the mediation effect analysis, professional identity threat was added as a mediating variable in model 2 and framed two paths: (1) loss of skill → professional identity threat → cyberloafing and (2) loss of autonomy → professional identity threat → cyberloafing. Table 8 shows that the loss of skill (β = 0.231, *p* < 0.001) and loss of autonomy (β = 0.326, *p* < 0.001) were positively related to professional identity threat. This means that employees fear losing their professional identity because of the loss of a sense of competence and control at their workplace due to AI. Hence, Hypotheses H2 and H3 are supported. Furthermore, professional identity threat significantly increased cyberloafing (β = 0.289, *p* < 0.001), suggesting that employees engage in cyberloafing to protect their threatened professional identity. 

When professional identity threat (PIT) was introduced as a mediator, the explained variance for cyberloafing increased to 32.1% (R^2^ = 0.321), and PIT itself was predicted by the loss of skill and loss of autonomy with an explained variance of 34.8% (R^2^ = 0.348). In this mediated model, the effects of the loss of skill (β = 0.179, *p* < 0.001) and loss of autonomy (β = 0.245, *p* < 0.001) on cyberloafing were reduced, while PIT demonstrated a significant positive effect on cyberloafing (β = 0.289, *p* < 0.001). The reduction in the direct path coefficients, coupled with the significant indirect effect through PIT (Table 9), supports the presence of partial mediation. These results suggest that the impact of the loss of skill and loss of autonomy on cyberloafing is partly explained by the extent to which such appraisals increase professional identity threat, which in turn fosters disengagement behaviors in the form of cyberloafing.

#### 4.6.3. Moderation Effects

**Figure 2:**
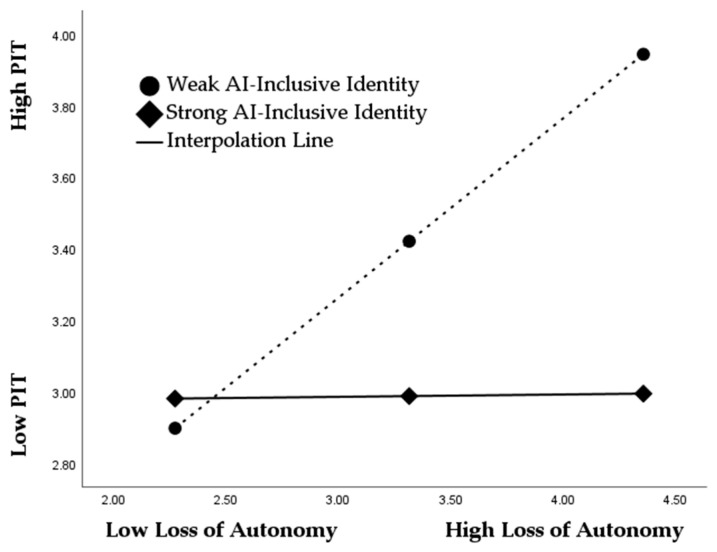
The moderated effect of AI-inclusive identity between the loss of autonomy and professional identity threat.

**Figure 3:**
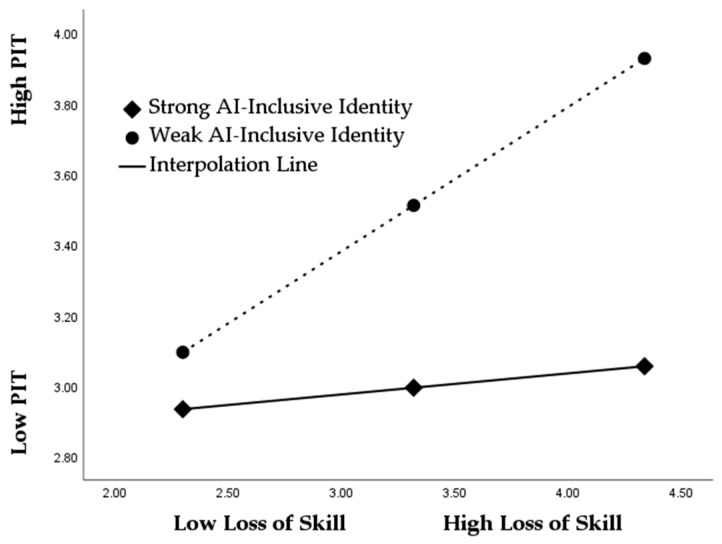
The moderated effect of AI-inclusive identity between the loss of skill and professional identity threat.

To examine moderation effects, AI-inclusive identity was incorporated into the model between independent variables and professional identity threat, with interaction terms (loss of expertise × AI-inclusive identity) and (loss of autonomy × AI-inclusive identity). The results illustrated in Table 10 show that the interaction effects are negative and statistically significant for both the loss of autonomy (β = −0.227, *p* < 0.001) and loss of skill (β = −0.153, *p* < 0.05). These findings demonstrate that AI-inclusive identity weakens the positive relationship between AI-driven appraisal threats and professional identity threat. In other words, employees who psychologically integrate AI into their professional self-concept are less likely to experience identity threat when facing autonomy or skill loss. Accordingly, H6 and H7 are supported. Further support for these hypotheses is provided by the slope diagrams (Figure 2 and Figure 3). In the case of the loss of autonomy, the simple slope for employees with weak AI-inclusive identity is steep and strongly positive, indicating that professional identity threat increases sharply as autonomy loss rises. In contrast, the slope for employees with strong AI-inclusive identity is nearly flat, suggesting that increases in autonomy loss lead to minimal changes in identity threat. A similar pattern emerges for the loss of skill: the slope is steep and upward under weak AI-inclusive identity but substantially flatter under strong AI-inclusive identity. The clear difference in slope steepness visually confirms the buffering effect of AI-inclusive identity, thereby reinforcing the statistical evidence and providing graphical validation for H6 and H7.

#### 4.6.4. Moderated Mediation Effects 

Table 11 presents the conditional indirect effects of the AI-driven loss of autonomy and loss of skill on cyberloafing through professional identity threat at different levels of AI-inclusive identity. The results reveal a clear moderated mediation pattern. For the loss of autonomy, the indirect effect is the strongest and highly significant when AI-inclusive identity is weak (β = 0.243, *p* < 0.001), decreases at the mean level (β = 0.128, *p* < 0.001), and becomes non-significant at high levels of AI-inclusive identity (β = 0.013, *p* = 0.583), as the confidence interval includes zero. This indicates that when employees strongly integrate AI into their professional identity, the indirect pathway from autonomy loss to cyberloafing via identity threat essentially disappears. A similar but slightly weaker pattern emerges for the loss of skill: the indirect effect is the strongest at low levels of AI-inclusive identity (β = 0.103, *p* = 0.001), remains significant at the mean level (β = 0.078, *p* < 0.001), and becomes substantially weaker though still significant at high levels (β = 0.052, *p* = 0.035). Overall, these findings demonstrate that AI-inclusive identity buffers the identity threat mechanism that links AI-driven appraisal threats to cyberloafing. Importantly, this moderated mediation pattern is fully consistent with the earlier moderation results, confirming that AI-inclusive identity reduces both the direct impact of autonomy and skill loss on professional identity threat and the subsequent indirect effect on cyberloafing. Hence, Hypotheses H8 and H9 are supported. 

## 5. Discussion

This study examined how AI collaboration reshapes employee behavior through identity processes. The findings reveal a coherent mechanism: AI-driven appraisals of autonomy loss and skill erosion undermine professional identity, which subsequently increases cyberloafing. Importantly, this identity-based pathway is significantly weakened among employees who develop a strong AI-inclusive identity. These results suggest that the behavioral consequences of AI implementation are not merely responses to technological complexity or workload demands but reflections of deeper disruptions to employees’ professional self-concept (Ashforth & Mael, 1989; Petriglieri, 2011).

Drawing on social identity theory, individuals derive meaning and self-worth from valued group memberships and professional roles (Ashforth & Mael, 1989; Ibarra, 1999). AI systems, particularly those capable of autonomous decision-making, may function as comparative team members rather than neutral tools (Raisch & Krakowski, 2021). When AI performs core tasks or constrains discretion, employees may interpret these changes as signals that their expertise is less central or valued. Such appraisals threaten identity continuity and legitimacy (Petriglieri, 2011), producing professional identity threat. Unlike emotional reactions such as anxiety or frustration often emphasized in technostress research (Ragu-Nathan et al., 2008; Tarafdar et al., 2019), identity threat challenges “who I am” in the workplace, thereby exerting stronger and more enduring behavioral influence.

The mediation results further clarify why cyberloafing emerges in AI-enabled environments. Prior research has framed cyberloafing as a response to stressors or resource depletion (Lim & Teo, 2005; Pindek et al., 2018). The present findings extend this perspective by suggesting that cyberloafing may function as an identity regulation behavior. When professional worth or autonomy is perceived as diminished, employees may engage in non-work-related online activities to symbolically reclaim control and self-direction. In this sense, cyberloafing becomes a behavioral response to perceived identity erosion rather than mere misconduct.

The moderation findings introduce an important boundary condition. AI-inclusive identity significantly weakens the translation of AI-driven threat appraisals into professional identity threat and weakens the subsequent indirect effect on cyberloafing. Identity integration processes have long been recognized as protective mechanisms that reduce intergroup threat and defensiveness (Ashforth & Schinoff, 2016). Employees who incorporate AI into their professional self-concept may reinterpret AI-imposed constraints as collaborative rather than competitive, thereby reducing perceived status threat. These results highlight that the psychological framing of AI (whether categorized as “us” or “them”) fundamentally shapes identity and behavioral outcomes.

### 5.1. Theoretical Contributions 

This study makes several important theoretical contributions to the literature on human–AI collaboration, professional identity, and employee behavior. First, this research advances the understanding of AI-enabled work by demonstrating that AI-driven changes are not merely technological disruptions but identity-relevant experiences that shape employees’ professional self-concept. Prior research on workplace technologies has predominantly emphasized technostress, cognitive overload, and emotional strain (Ragu-Nathan et al., 2008; Tarafdar et al., 2019). While these perspectives capture important affective reactions, they do not fully explain how AI systems, particularly those capable of autonomous decision-making, symbolically encroach upon domains central to professional expertise and judgment (Raisch & Krakowski, 2021). Drawing on social identity theory (Ashforth & Mael, 1989) and identity threat logic (Petriglieri, 2011), this study shows that the perceived loss of autonomy and skill erosion operate through professional identity threat. In doing so, it reconceptualizes AI collaboration as a process that directly engages and potentially destabilizes employees’ role-based identity, thereby extending identity theory into the domain of intelligent technologies.

Second, this study clarifies the psychological mechanism linking AI-driven appraisals to disengagement behavior. Although prior research has linked technological change to outcomes such as anxiety, exhaustion, and stress (Ragu-Nathan et al., 2008; Tarafdar et al., 2019), fewer studies have isolated identity-based processes as the central explanatory pathway. By empirically establishing professional identity threat as the mediator between the AI-driven loss of autonomy and skill and cyberloafing, this study differentiates identity disruption from general affective strain. Identity threat represents a challenge to professional legitimacy and self-worth (Petriglieri, 2011), which may produce behavioral responses distinct from those driven by temporary emotional states. This theoretical distinction deepens our understanding of why AI-related changes can result in withdrawal behaviors even when objective task demands remain manageable.

Third, the introduction of AI-inclusive identity as a boundary condition refines identity regulation models in AI-enabled workplaces. Prior research suggests that identity integration processes can reduce perceived intergroup threat and defensiveness (Ashforth & Schinoff, 2016). Consistent with this view, our findings demonstrate that when employees incorporate AI into their professional self-concept, the positive relationship between AI-driven threat appraisals and professional identity threat weakens, and the indirect pathway to cyberloafing diminishes. Rather than treating identity orientations as static traits, this study shows that AI-inclusive identity reshapes how employees categorize AI by transforming it from a perceived outgroup competitor into an ingroup collaborator. 

Finally, this study contributes to the literature on counterproductive and withdrawal behaviors by reconceptualizing cyberloafing as an identity-relevant behavioral outcome in AI-enabled environments. Prior research has often framed cyberloafing as deviant behavior or a response to job stressors (Lim & Teo, 2005; Pindek et al., 2018). By linking cyberloafing to professional identity threat, this study demonstrates that disengagement behaviors may arise not simply from workload or strain but from the perceived erosion of professional meaning in the face of AI-driven changes. This perspective broadens theoretical understanding of employee disengagement in digital and algorithmically managed workplaces (Derks et al., 2015; Lim & Chen, 2012).

### 5.2. Practical Contributions 

First, organizations implementing AI systems should recognize that the perceived loss of autonomy and skill relevance can undermine employees’ professional identity. These perceptions are not merely operational concerns but psychological signals of role displacement (Petriglieri, 2011). Managers should therefore assess not only productivity outcomes but also employees’ identity-related perceptions during AI integration processes.

Second, AI systems should be designed and communicated as augmentative rather than substitutive technologies. Preserving human discretion, maintaining override authority, and clearly defining complementary roles can reduce the perceptions of professional displacement (Raisch & Krakowski, 2021). Transparent communication regarding the collaborative purpose of AI may prevent identity threat from emerging.

Third, fostering AI-inclusive identity is essential. Training programs should go beyond technical skill development and emphasize psychological integration, helping employees view AI as part of their professional team. Participatory design processes and collaborative framing can reduce perceived intergroup threat and facilitate adaptive identity adjustment (Ashforth & Schinoff, 2016).

Finally, managers should interpret cyberloafing cautiously in AI-enabled environments. Rather than responding solely with monitoring and sanctions, organizations may benefit from identifying whether disengagement behaviors reflect underlying identity concerns. Addressing identity misalignment may prove more effective than intensifying algorithmic control.

### 5.3. Limitations and Future Research Agenda 

This study has several limitations that point to meaningful directions for future research. First, although the three-wave time-lagged design reduces concerns about common method bias, the data are based on self-reported perceptions. Employees’ appraisals of AI-driven autonomy and skill loss and cyberloafing behaviors may be influenced by subjective interpretations. Future studies could incorporate multi-source data, such as supervisor evaluations, peer reports, or objective system-logged indicators of online behavior, to strengthen causal inference.

Second, this study focuses on professional identity threat as the central psychological mechanism linking the AI-driven loss of autonomy and skill to cyberloafing. While identity threat is theoretically appropriate in AI-enabled work, other psychological mechanisms, such as anger, anxiety, emotional exhaustion, or work alienation, may also explain employee disengagement. Future research should use parallel or competing mediation models to compare identity-based mechanisms with affective and resource-based pathways, thereby clarifying when and why identity threat is the most salient bridge between AI appraisal and behavior.

Third, although we argue that AI-driven threats differ from those associated with earlier technologies, this study does not empirically compare AI with traditional IT or automation systems. Future research could adopt comparative designs to examine whether AI’s autonomous, decision-making, and substitutive features uniquely intensify identity threat beyond what is observed with prior technological innovations. Such comparisons would further establish the distinctiveness of AI-related identity processes.

Fourth, AI-inclusive identity was treated as a relatively stable moderator in this study. However, employees’ identification with AI is likely to evolve over time as they gain experience, training, or exposure to intelligent systems. Longitudinal or experience-sampling studies could explore how AI-inclusive identity develops dynamically and whether it shifts from a buffering resource to a potential source of tension under prolonged or intensified AI use.

Finally, this study was conducted within a single cultural and organizational context, which may limit the generalizability of the findings. Cultural norms related to autonomy, authority, and technology acceptance may shape how employees interpret AI-driven changes. Future research should examine these relationships across different cultural, occupational, and institutional settings and explore additional boundary conditions such as leadership style, ethical AI practices, or job design characteristics.

## Figures and Tables

**Table 1:** Comparison between previous studies.

Theoretical Lens	Typical Explanatory Path (Simplified)	Implications	Limitation	References
COR/resource depletion	Stressor → resource loss/exhaustion → cyberloafing	Cyberloafing as response to depletion; mediating role of emotional exhaustion	Treats tech/AI changes as generic stressors; does not theorize identity-relevant meaning	Hobfoll (1989); Hobfoll et al. (2018); Lim and Teo (2005); Koay and Soh (2018); Lu et al. (2024)
Coping/avoidance framing	Stressor → negative affect → avoidant coping (cyberloafing)	Cyberloafing as short-term psychological escape	Often underspecifies what is threatened (tasks vs professional self)	Lazarus and Folkman (1984); Lim and Chen (2012); Koay and Soh (2018); S. Zhang et al. (2024)
Algorithmic management/surveillance and control	Algorithmic monitoring → lower autonomy → resistance/worse outcomes	Establishes autonomy loss as core experience under algorithmic systems	Does not explain why autonomy loss becomes professional identity threat nor why it yields specific coping behavior	Kellogg et al. (2020); Möhlmann and Zalmanson (2017); Rosenblat and Stark (2016); Parent-Rocheleau and Parker (2022)
Digital transformation	Skill obsolescence viewed as threat appraisal → identity threat	Identifies AI-induced appraisals that cause identity threat	Lacks linking identity threat to any responsive behavior or outcome	(Mirbabaie et al., 2022)
Digital transformation resistance (threat-based)	Digital tech viewed as threat → resistance/negative emotions	Catalogs “threat perceptions” in digital transformation; acknowledges emotion	Often broad (job security/resources); limited micro-mechanism specifying professional identity threat from skill/autonomy losses	Lapointe and Rivard (2005); Kim and Kankanhalli (2009); Vial (2019); Verhoef et al. (2021)
Identity threat/SIT-based mechanism	Skill loss + autonomy loss (appraisals) → professional identity threat → coping (cyberloafing)	Explains why tech change is existentially threatening; predicts both strain and self-protective coping behaviors	Needs to incorporate other digital variations and integration with the cyberloafing literature	This study

**Table 2:** Demographics of participants.

Demographics	Items	Frequency	Percentage
Gender	Male	230	45.5%
	Female	277	54.6%
Age	Below 30	238	46.9%
	31–40	167	32.9%
	41–50	58	11.4%
	Above 50	44	8.7%
Years of employment	1–3	25	4.9%
	4–6	125	24.7%
	7–9	110	21.7%
	10–12	137	27.0%
	More than 10 years	110	21.7%

**Table 3:** Confirmatory factor analysis.

Models	χ^2^	df	RMSEA	SRMR	CFI	TLI	IFI
five-factor model LA, LS, PIT, CF, AID	1270.803	314	0.078	0.077	0.901	0.899	0.869
four-factor model LA + LS, PIT, CF, AID	1683.168	318	0.092	0.101	0.817	0.798	0.818
three-factor model LA + LS, PIT + CF, AID	2384.989	321	0.113	0.132	0.724	0.698	0.725
two-factor model LA + LS + PIT + CF, AID	2794.948	323	0.123	0.138	0.669	0.641	0.671
one-factor model LA + LS + PIT + CF + AID	3582.612	324	0.141	0.159	0.564	0.528	0.566

**Table 4:** Reliability and validity of constructs.

Construct	Items	Loadings	α	CR	AVE
Professional identity threat (PIT)	TR1	0.831	0.885	0.916	0.686
	TR2	0.820			
	TR3	0.824			
	TR4	0.845			
	TR5	0.820			
	TC1	0.809	0.917	0.934	0.667
	TC2	0.806			
	TC3	0.822			
	TC4	0.819			
	TC5	0.820			
	TC6	0.822			
	TC7	0.822			
Loss of skill (LS)	LS1	0.877	0.848	0.908	0.767
	LS2	0.865			
	LS3	0.885			
Loss of autonomy (LA)	LA1	0.861	0.833	0.900	0.750
	LA2	0.858			
	LA3	0.879			
AI-inclusive identity (AID)	AID1	0.842	0.881	0.913	0.677
	AID2	0.838			
	AID3	0.828			
	AID4	0.823			
	AID5	0.827			
Cyberloafing	CF1	0.810	0.866	0.909	0.714
	CF2	0.855			
	CF3	0.860			
	CF4	0.853			

**Table 5:** Discriminant validity Fornell–Larcker criterion.

	AID	LS	PIT	CF	LS
AID	0.823				
LA	−0.417	0.866			
PIT	−0.458	0.508	0.848		
CF	−0.317	0.461	0.486	0.845	
LS	−0.351	0.432	0.437	0.404	0.876

**Table 6:** Heterotrait–monotrait (HTMT) ratio.

	AID	LA	PIT	CF	LS
AID					
LA	0.486				
PIT	0.623	0.711			
CF	0.364	0.540	0.667		
LS	0.406	0.513	0.607	0.470	

**Table 7:** Descriptives and correlation coefficients.

	Mean	Std. Deviation	LS	LA	AID	CF	PIT
**LS**	3.3176	1.018	1	−0.041	0.090	0.403 ***	0.439 ***
**LA**	3.3149	1.040		1	0.141	0.459 ***	0.503 ***
**AID**	3.3925	0.984			1	−0.166 *	0.107 *
**CF**	3.3555	0.971				1	−0.483 ***
**PIT**	3.3128	0.822					1

***: Correlation at 0.001 (2-tailed). *: Correlation is significant at the 0.05 level (2-tailed).

**Table 8:** Results of main and mediation analyses.

	Model 1		Model 2			
	DV:Cyberloafing		DV:PIT		DV:Cyberloafing	
Variables	β	*t*-Value	β	*t*-Value	β	*t*-Value
*Control variables*						
Gender	0.074	0.912	0.021	0.275	0.068	0.865
Age	−0.027	0.263	0.119	1.206	−0.062	0.679
Tenure (years)	0.064	0.649	0.088	0.918	0.040	0.429
*Independent variables*						
Loss of autonomy	0.340 ***	7.795	0.326 ***	7.960	0.245 ***	5.263
Loss of skill	0.246 ***	5.849	0.231 ***	5.758	0.179 ***	4.231
*Mediator*						
PIT					0.289 ***	6.216
R^2^	0.268		0.348		0.321	

*** *p* < 0.001.

**Table 9:** Specific indirect effects.

Paths	β	Boot SE	*t*	*p*	95% CI
LA → PIT → CF	0.187	0.026	7.139	0.000	[0.138, 0.242]
LS → PIT → CF	0.132	0.022	5.917	0.000	[0.090, 0.178]

**Table 10:** Moderation effect analysis.

	DV:PIT	
Variables	β	*t*-Value
*Control variables*		
Gender	0.000	0.023
Age	0.089	0.955
Tenure (years)	0.038	0.414
*Independent variables*		
Loss of autonomy	0.240 ***	5.576
Loss of skill	0.144 ***	3.484
*Moderator*		
AI-inclusive identity	0.101	1.368
*Interaction term*		
Loss of autonomy × AI-inclusive identity	−0.227 ***	6.530
Loss of skill × AI-inclusive identity	−0.153 *	2.464
R^2^	0.448	

* Indicates statistical significance at the 10% level, *** indicates statistical significance at the 1% level.

**Table 11:** Conditional indirect effects of AI-driven loss of autonomy and loss of skill on cyberloafing via professional identity threat.

Pathway	AI-Inclusive Identity	β	Boot SE	*t*-Value	*p*-Value	95% Bootstrap CI
LA → PIT → CF	Low (−1 SD)	0.243	0.036	6.828	0.000	[0.177, 0.316]
	Mean	0.128	0.024	5.246	0.000	[0.084, 0.179]
	High (+1 SD)	0.013	0.024	0.550	0.583	[−0.032, 0.064]
LS→ PIT → CF	Low (−1 SD)	0.103	0.030	3.470	0.001	[0.049, 0.164]
	Mean	0.078	0.021	3.683	0.000	[0.039, 0.122]
	High (+1 SD)	0.052	0.025	2.109	0.035	[0.006, 0.103]

## Data Availability

The datasets used in this research are available upon request from the corresponding author. The data are not publicly available due to restrictions, i.e., privacy or ethical.
